# Enhanced nasal drug delivery efficiency by increasing mechanical loading using hypergravity

**DOI:** 10.1038/s41598-017-18561-x

**Published:** 2018-01-09

**Authors:** Dongjoo Kim, Young Hyo Kim, Soonjo Kwon

**Affiliations:** 1 WCSL of Integrated Human Airway-on-a-chip, Inha University, Incheon, 22212 Korea; 2 Department of Biological Engineering, Inha University, Incheon, 22212 Korea; 3 Department of Otorhinolaryngology, Inha University College of Medicine, Incheon, 22332 Korea

## Abstract

Nasal route drug administration for local and systemic delivery of many therapeutics has received attention because the nasal cavity is highly vascularized and provides a large surface area for drug absorption. However, nasal mucosa exhibits limited permeability to polar molecules. In this study, we developed a novel method for improving absorption efficiency of polar drugs by applying hypergravity. RPMI 2650 cells and primary human nasal epithelial cells were exposed three times to a 20 min hypergravitational condition (10 × *g*) with a 20 min rest period after each exposure. The applied hypergravity induced a decrease in transepithelial electrical resistance without significant loss of cellular metabolic activity, and cellular permeability of fluorescein sodium salt (MW 376 Da; NaFI) and FITC-labeled dextran (average MW 4,000 Da; FD-4) increased by 19% and 16%, respectively. Immunostaining and RT-qPCR results demonstrated that hypergravity conditions affected cytoskeletal structures and tight junctions, leading to weakening of the cell barrier function and increasing the cellular permeability of polar molecules. Our results indicate that hypergravity could be used as a new strategy for enhancing the efficiency of drug absorption via the nasal route.

## Introduction

Needle insertions cause pain, result in scar tissues in patients who suffer from chronic diseases, and induce needle phobia in child patients, thus the demand for needle-free drug delivery techniques has been increasing. There are several strategies for needleless drug delivery systems, such as hypodermic injection using jet injectors, microneedle for transdermal injection, and intranasal administration^1–5^. Among them, intranasal drug delivery is a commonly used needle-free drug delivery technique. The nasal route is simple, convenient, non-invasive, reliable, and safe for systemic administration of drugs^6,7^. Further advantages for systemic drug delivery via the nasal route include its large surface area for drug absorption and the associated avoidance of hepatic first-pass metabolism and drug degradation in gastrointestinal tract^8–10^. Moreover, nasal route drug delivery is an attractive strategy for brain targeted drug delivery because it allows therapeutic agents to be delivered to the brain via neural pathways such as the olfactory and trigeminal nerves, thereby bypassing the blood-brain barrier^11^. Thus, intranasal administration can be an effective alternate technique to drug delivery via needle insertion, and it can encourage the treatment of brain disorders.

Drugs can cross the nasal mucosa by two different routes; the transcellular (across the cell membrane) and paracellular (between the cells) pathways^9,12,13^. Lipophilic molecules are generally satisfactorily transported via the transcellular pathway including receptor-, carrier-, and vesicle-mediated routes^12,14^. Polar molecules are generally considered to cross the epithelium via gaps between cells; however, such molecules, particularly large molecular weight peptides and proteins, exhibit limited permeability in nasal mucosa^14,15^. Several strategies have been developed to improve the cellular transport rate of polar drugs, and many strategies involve absorption-promoting agents such as surfactants, bile salts (or their derivatives), phospholipids, cyclodextrins, and cationic polymers^16–20^. However, those enhancers have toxic potentials such as the capability to cause severe morphological alterations, significant membrane damage in the epithelium, and inhibition of mucociliary transport^21,22^.

**Figure 1:**
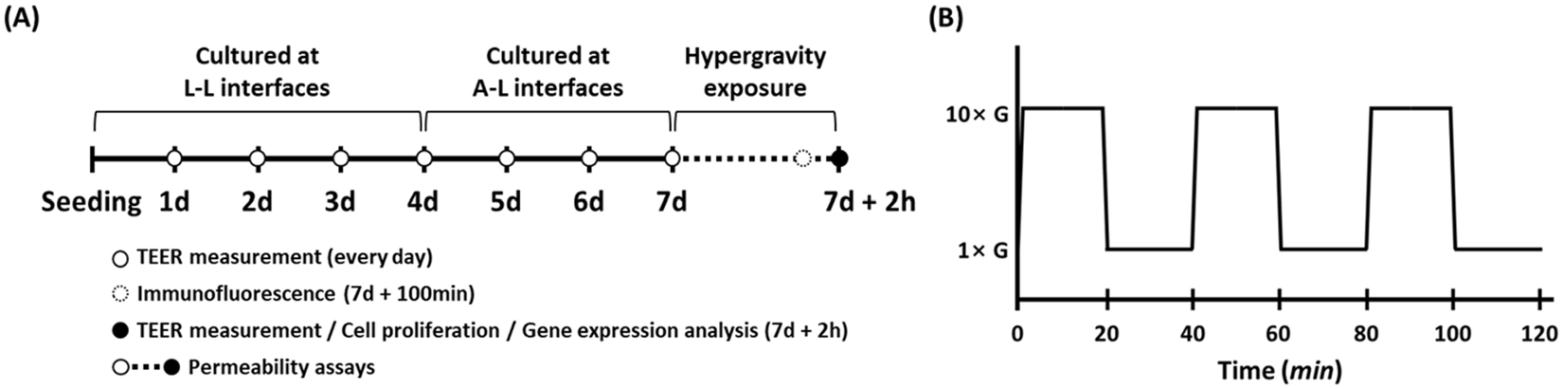
(**A**) Schematic diagram for the cell culture and hypergrvity exposure process. RPMI 2650 cells were cultured at liquid-liquid (L-L) interfaces for 4 days, then at air-liquid (A-L) interfaces for 3 days. Each hypergravity exposure was performed as indicated below. (**B**) Schematic diagram for hypergravity exposure, which entailed three 20 min periods of hypergravity exposure, each followed by a 20 min rest period.

In this study, we used hypergravity in a novel strategy for enhancing nasal drug delivery efficiency. Previous studies have suggested that hypergravity exposure can alter cell barrier function by downregulation of occludin expression, decrease of transendothelial electrical resistance, and increase of paracellular permeability in human umbilical vein endothelial cells^23,24^. Because the cell barrier function is closely related to cellular drug uptake, we hypothesized that mechanical load increase by hypergravity exposure could be utilized for improving nasal drug delivery efficiency. To test this hypothesis, a 10-fold gravitational force was applied to human nasal epithelial cell line (RPMI 2650) and primary human nasal epithelial (hNE) cells by using a centrifuge (Fig. 1). We investigated the effects of the applied hypergravity on transepithelial electrical resistance (TEER), cell proliferation, cellular permeability, cytoskeletal structures, and gene expressions. The hypergravitational force weakened cell barrier function and increased the cellular permeability of polar molecules. Further, cytoskeletal structures were altered and gene expression levels of junctional proteins and β-actin were upregulated following exposure to hypergravity.

## Results

### Cell proliferation

**Figure 2:**
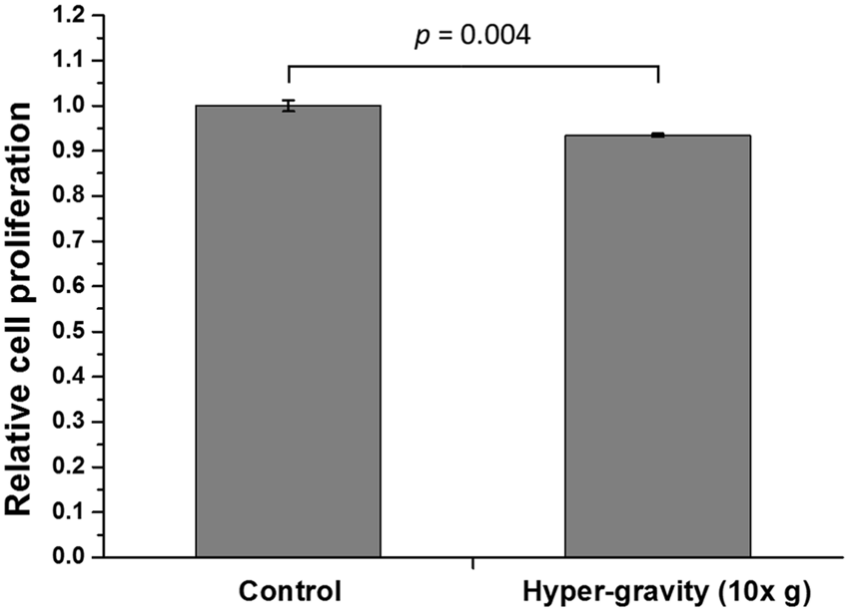
Cell proliferation of RPMI 2650 cells following exposure to the hypergravity stimulation protocol. The relative cell proliferation ratio was calculated by normalizing the absorbance of the hypergravity exposed group to that of the control group (*n* = 3).

A cell counting kit-8 (CCK-8) assay was used to investigate the hypergravitational effect on the metabolic activity of RPMI 2650 cells (Fig. 2). The applied hypergravity induced a slight, but statistically significant decrease in cell proliferation. Metabolic activity of the test group was 6.5% lower than that of the control group (*p* < 0.01).

### TEER measurements

**Figure 3:**
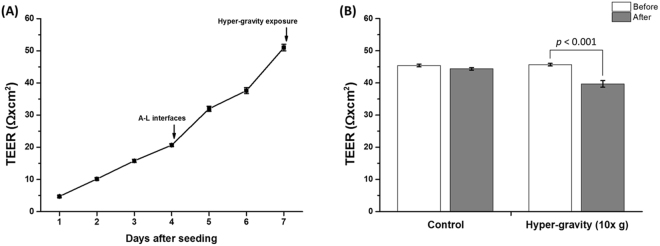
The TEER values across RPMI 2650 cell layers. (**A**) The TEER values were monitored for 7 days (*n* = 42). The A-L interfaces were formed 4 days after seeding, and the hypergravity protocol was applied 3 days after A-L interfaces formation. (**B**) Effects of hypergravity on TEER values in RPMI 2650 cell layers (*n* = 3).

TEER values of the RPMI 2650 cell layers were determined to monitor cell barrier function. As shown in Fig. 3A, TEER values increased to 20 Ω × cm^2^ under the liquid-liquid (L-L) condition, whereas they rapidly increased and reached 50 Ω × cm^2^ under the air-liquid (A-L) condition. We also measured TEER before and after hypergravity exposure to investigate the effect of hypergravitational forces on cell barrier function (Fig. 3B). Before hypergravity exposure, the control and test groups had TEER values of 45.4 and 45.7 Ω × cm^2^, respectively. TEER of the test group decreased significantly to 39.7 Ω × cm^2^ (*p* < 0.001) after completion of the 2 hour hypergravity stimulation protocol, while that of control group did not significantly change.

### Permeability assays

To investigate the effect of hypergravity on cellular permeability, RPMI 2650 cell layers were established on permeable transwell inserts, after which the apparent permeability (*P*_*app*_) values of fluorescein sodium salt (NaFI), FITC-labeled dextran (FD-4), and fluorescein-labeled jacalin were obtained (Table 1). The applied hypergravitational forces altered the permeation rate of RPMI 2650 cell layers for NaFI and FD-4. Mean *P*_*app*_ of the control group was 5.429 × 10^−6^ ± 0.081 × 10^−6^ cm/s for NaFI and 2.093 × 10^−6^ ± 0.010 × 10^−6^ cm/s for FD-4, while those of the hypergravity exposed group were significantly higher at 6.465 × 10^−6^ ± 0.108 × 10^−6^ cm/s for NaFI (*p* < 0.01) and 2.427 × 10^−6^ ± 0.058 × 10^−6^ cm/s for FD-4 (*p* < 0.01). There was no significant difference in the mean *P*_*app*_ of fluorescein-labeled jacalin between the control (0.756 × 10^−6^ ± 0.091 × 10^−6^ cm/s) and test (0.774 × 10^−6^ ± 0.158 × 10^−6^ cm/s) groups.

**Table 1:** The apparent permeation coefficients (10^−6^ cm/s) of NaFI, FD-4, and fluorescein-labeled jacalin through RPMI 2650 cell layers.

Target marker	Control	Hypergravity	Cell-free transwell insert
NaFI	5.429 ± 0.081	6.465 ± 0.108 (*p* = 0.00154)	15.356 ± 0.348
FD-4	2.093 ± 0.010	2.427 ± 0.058 (*p* = 0.00471)	7.942 ± 0.300
Fluorescein-labeled jacalin	0.756 ± 0.091	0.774 ± 0.158	2.572 ± 0.016

### Immunofluorescence

**Figure 4:**
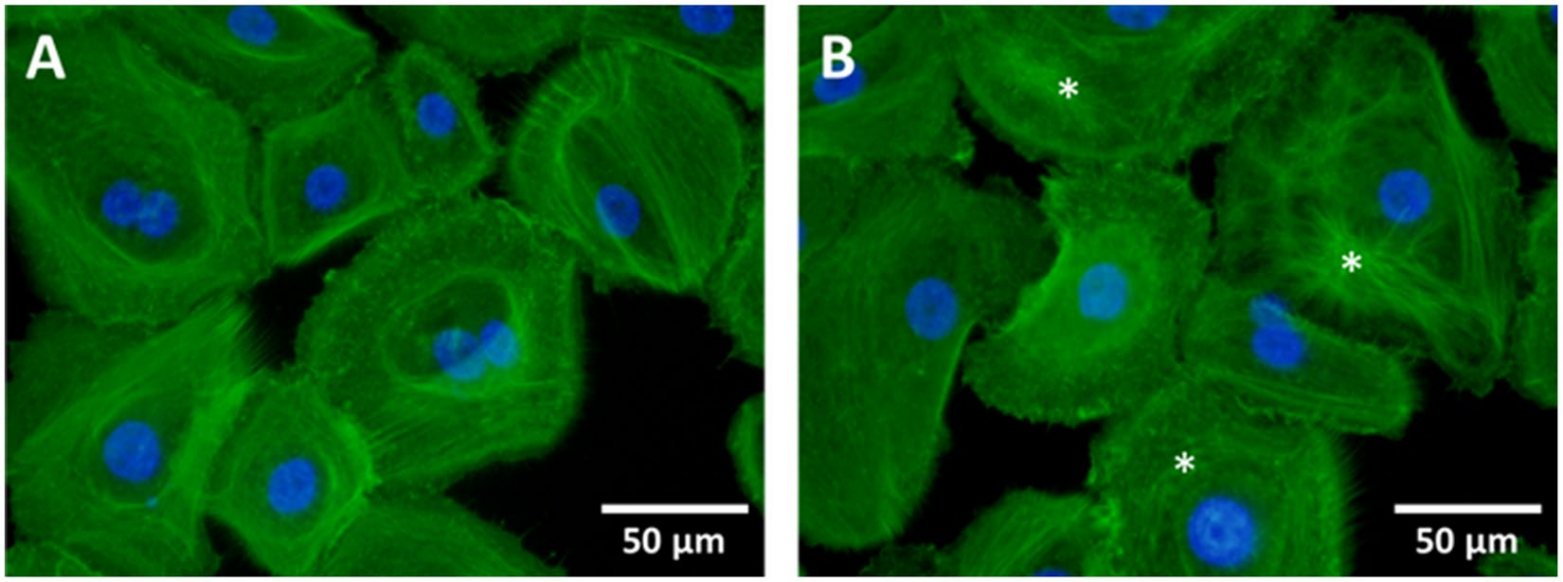
Immunofluorescence images of F-actin with AlexaFluor 488 conjugated phalloidin and DAPI for counterstaining in hNE cells. (**A**) Static control group; (**B**) hypergravity exposed group. Asterisks indicated rearranged (or deformed) cytoskeletal structures.

Immunofluorescence analyses were performed to determine the hypergravitational effects on the cytoskeletal structures in hNE cells. Structural differences in cytoskeletons were observed between the static control and hypergravity exposed groups (Fig. 4). Actin filaments encircled the nucleus and were more distinctly visualized in control group cells than in cells exposed to hypergravity (Fig. 4A). Following completion of the 2 hour hypergravity stimulation protocol, cytoskeletal structure arrangement was altered and immunofluorescence intensities decreased (Fig. 4B).

### Gene expression analysis

**Figure 5:**
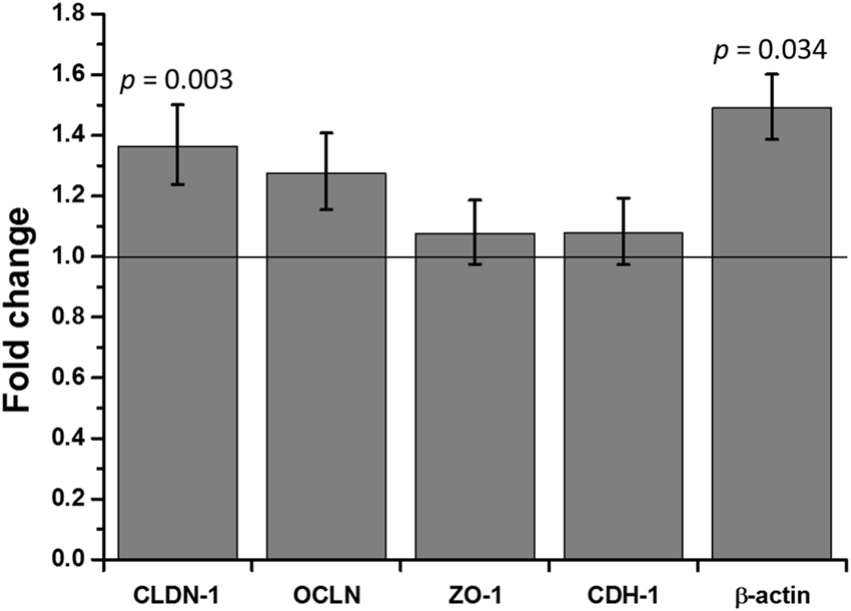
Fold changes in gene expression levels of junctional proteins in RPMI 2650 cells after applying the hypergravity protocol. The relative gene expression level was calculated by using the 2^(−ΔΔCT)^ method, and levels were normalized by the GAPDH housekeeping gene and the static control (*n* = 3).

Gene expression levels of claudin-1 (CLDN-1), occludin (OCLN), zonula occludens-1 (ZO-1), cadherin-1 (CDH-1), and β-actin were investigated by performing real-time quantitative PCR (RT-qPCR) (Fig. 5). Among the tight junctions related genes, the expression level of CLDN-1 was 1.36-fold higher in the hypergravity exposed group than in the control group (*p* < 0.01). The expression of OCLN also increased by 27%, but it was not statistically significant. There was no remarkable change in the ZO-1 expression. In the case of CDH-1, which has important roles in cell adhesion, its expression level was also not changed following hypergravity exposure. However, β-actin gene expression increased by 49% (*p* < 0.05) following exposure of hypergravity.

## Discussion

The nasal cavity can be divided into three distinct areas; the vestibule, the olfactory region, and the respiratory zone^25,26^. The vestibule area filters aerodynamic particles larger than 10 μm in inhaled air, and the olfactory region has an important role in olfactory perception. The respiratory zone can be divided into superior, middle, and inferior turbinate areas. These turbinates create airflow turbulence, which is important in humidifying and warming the inhaled air. Because the nasal cavity is highly vascularized and has a large surface area, it has been regarded as an optimal site for drug absorption^27^. However, delivery of candidate drugs through the nasal pathway is generally limited to lipophilic molecules. Although several methods have been developed for enhancing the absorption efficiency of polar drugs, those methods also have side effects. Thus, we developed a novel strategy to increase the delivery efficiency of polar drugs by increasing mechanical loading using hypergravity conditions. Even though hypergravity conditions are generally thought to be harmful and noxious to the human, there have been studies indicating beneficial effects of hypergravity on living organisms, such as enhancing differentiation of bone marrow mesenchymal stem cells into cardiomyocytes and neuronal cells, upregulating osteopontin expression in osteoblasts, and increasing immune functions in animals with allergic disorders^28–31^. In addition, Sumanasekera *et al*. reported that exposure to hypergravity can induce a change in cell barrier function in endothelial cells^23,24^. In this study, we hypothesized that hypergravity could be used as a novel strategy for improving drug delivery efficiency. To assess our hypothesis, RPMI 2650 cells and hNE cells were exposed to 10 × *g* hypergravity conditions to determine its effects on cell barrier function. Our results showed that the applied hypergravity increased cellular permeability of NaFI and FD-4 in RPMI 2650 cell layers and reduced the TEER value related to cell barrier function. We also observed that hypergravity upregulated gene expressions of some junctional proteins and β-actin in RPMI 2650 cells. Further, cytoskeletal structures were altered after hypergravity exposure.

RPMI 2650 cell line has shown consistent growth, and high stability *in vitro* without loss of normal diploid karyotype^32,33^. Further RPMI 2650 cells form a cellular barrier with junctional proteins, including ZO-1, OCLN, CLDN-1, CDH-1, and β-catenin^34,35^. They have been used as an *in vitro* model for nasal permeation studies in recent years, reporting that RPMI 2650 cells cultured at A-L conditions express sufficient barrier properties such as high TEER values and permeation coefficients^34,36,37^. Thus, we also used RPMI 2650 cells as an *in vitro* nasal model and evaluated TEER and *P*_*app*_ values of NaFI, FD-4, and fluorescein-labeled jacalin. Since NaFI and FD-4 do not show significant toxic effects and have fluorescence properties which enable more sensitive detection of subtle changes, they have been widely used as tracers in permeability assays^38^. In case of marker for adsorptive endocytosis, the lectin jacalin have strong binding affinity to the Thomsen-Friedenreich antigen located on cell membrane, thus it could be used as a membrane marker for adsorptive endocytosis^37^. Therefore NaFI, FD-4 and fluorescein-labeled jacalin were chosen as markers for paracellular permeation of small polar molecules, large polar molecules, and adsorptive endocytosis. In this study, RPMI 2650 cells cultured at A-L interfaces had TEER values of 50.98 Ω × cm^2^, and their *P*_*app*_ values were 5.429 × 10^−6^, 2.093 × 10^−6^, and 0.756 × 10^−6^ cm/s for NaFI, FD-4, and fluorescein-labeled jacalin, respectively. Our results are similar to those of previous studies with only slight differences detected, which may result from differences in culture conditions such as culture media and period.

There have been several studies into gravitational effects on cell proliferation. Tschopp and Cogoli reported that cell proliferation of various cell types cultured in a hypergravitational field (10 × *g*) increased by 20–30%^39^. In addition, Koyama *et al*. reported that hypergravity (3 × *g*) improved cell proliferation in bovine aortic endothelial cells^40^. In contrast, our results showed that the applied hypergravity (10 × *g*) decreased the proliferation rate only slightly (approximately 6.5%) in RPMI 2650 cells. These differences may be due to differences in hypergravity levels, cell lines, and culture conditions.

It is well known that TEER values depend on the expression of specific tight junction proteins such as CLDN-1, OCLN, and ZO-1^41^. Because tight junction proteins control the cell barrier function, TEER values can be used to assess the barrier functions of epithelial and endothelial cells^42^. In this study, hypergravity led to a 13% reduction of TEER values in RPMI 2650 cell layers. This result indicates that the cell barrier function of the RPMI 2650 cell layers was weakened by increasing mechanical loading using hypergravity conditions^43^. The weakened cell barrier function also led to changes in the permeation rate of hydrophilic molecules. We observed that the permeability of NaFI and FD-4 increased by 19% and 16%, but there was no change in the permeability of fluorescein-labeled jacalin. These results suggest a potential applicability of hypergravity for enhancing nasal drug delivery efficiency.

In the current study, the gene expression level of CLDN, OCLN, ZO-1, CDH-1, and β-actin were analyzed following exposure of cells to hypergravity. Our results showed that gene expressions of CLDN, OCLN and β-actin were upregulated following hypergravity exposure, but there were no significant changes in the expressions of ZO-1 and CDH-1. In addition, hypergravity induced structural changes in cytoskeleton. Adherens junction regulates actin cytoskeleton and leads to assembly of the tight junction, while tight junction proteins, such as CLDN and OCLN, bind to cytoplasmic proteins that interact with the actin cytoskeleton^44^. Versari *et al*. reported that hypergravity induced a reversible alteration of actin fiber distribution in endothelial cells^45^. In addition, Koyama *et al*. suggested that hypergravity caused transient reorganization of cytoskeleton via tyrosine phosphorylation and RhoA activation in bovine endothelial cells^40^. Considering our results and those in previous studies, we assume that the hypergravity condition applied in this study altered cytoskeletal arrangement, leading to deformation of tight junctions. Consequently, the process resulted in a weakened barrier function in RPMI 2650 cells. The increased expression level of junctional proteins and β-actin may be a physiological reaction to cytoskeleton, adherens junction, and tight junction reorganization.

In this study, we investigated the effect of hypergravity on cellular permeability and evaluated the applicability of hypergravity for improving nasal absorption of polar drugs. We observed that the applied hypergravity protocol enhanced the permeation rate of hydrophilic molecules in RPMI 2650 cell layers with only a slight decrease (6.5%) in cell proliferation. The results appear to be due to the altered arrangement of actin filament and junction proteins. Although further studies are needed to ensure its usefulness as a medical application, our results suggest that hypergravity could be used as a novel strategy for enhancing nasal drug delivery efficiency through temporal weakening of the cell barrier function.

## Materials and Methods

### Cell culture

The RPMI 2650 cells were obtained from the Korean Cell Line Bank (KCLB; Seoul, Korea). They were routinely cultured in T75 flasks at 37 °C with 5% CO_2_. Dulbecco’s modified Eagle’s medium (DMEM; Gibco, Carlsbad, CA, USA) supplemented with 10% FBS (Gibco) and 1% penicillin-streptomycin (Gibco) was used as the culture medium. For hypergravity exposure, RPMI 2650 cells (passage 48) were trypsinized from a T75 flask and seeded onto 24 mm Transwell filter inserts (CT3450, Corning, NY, USA) at a density of 300,000 cells/well. They were cultured at L-L interfaces for 4 days, then at A-L interfaces for 3 days to form tight barriers. For the L-L interface culture, 1.5 mL and 2.6 mL of culture media were added to the apical and basolateral side, respectively. For the A-L interface culture, media were removed from the apical side. Culture media were changed every 2 days.

We obtained human nasal inferior turbinate tissue samples from patients who underwent turbinectomy due to chronic rhinitis. All methods were performed in accordance with relevant guidelines and regulations, and all experimental protocols were approved by the Inha College of Medicine Institutional Review Board (INHAUH 2017-03-018-001). During the consent process, we obtained written informed consent and discussed with participants the potential risks of participation, including the physical risks associated with specimen collection and the possibility that protected health information or de-identified project data stored in a public repository could be accidentally released. The protocol and consent form described the precautions taken to reduce these risks. The hNE cells were isolated from the tissue samples by using a primary explant technique. Contaminating fibroblasts were removed by repeated scraping and differential trypsinization^46^. Isolated hNE cells were routinely cultured in T25 flasks coated with type I collagen (Gibco) at 37 °C with 5% CO_2_. Keratinocyte serum-free medium (K-SFM; Gibco) containing bovine pituitary extract (30 μg/mL; Gibco), human recombinant epidermal growth factor (5 ng/mL; Gibco), and gentamicin (5 μg/mL; Sigma, St Louis, MO, USA) was used as the culture medium, which was changed every 2 days. For hypergravity exposure, hNE cells (passages 3 and 4) were trypsinized and seeded onto 4.55 cm^2^ cell culture slides (SPL Life Science, Gyeonggi-do, Korea) at a density of 30,000 cells/cm^2^ and cultured for 1–2 days.

### Hypergravity exposure

Cell culture plate holder (HM-2, Hanil, Seoul, Korea) was installed in a conventional centrifuge (Fleta 5, Hanil). This centrifuge was used to apply a hypergravity condition of 10 × *g*. The RPMI 2650 cells were exposed three times to 20 min of the hypergravity condition with 20 min rest periods following each exposure (Fig. 1). The RPMI 2650 cells were maintained in A-L interfaces during hypergravity stimulation, except during permeability assays. The hNE cells were also exposed to same hypergravity stimulation protocol but without the final 20 min rest period. Controls of both RPMI 2650 cells and hNE cells were cultured separately without hypergravity exposure.

### Cell proliferation

A CCK-8 (Dojindo Laboratories, Kumamoto, Japan) was used to determine the effects of hypergravity on cell proliferation. Following completion of the 2 hour hypergravity stimulation protocol, the culture medium on the basolateral side was removed and 100 μL of CCK-8 solution were added to the apical side. After a one hour incubation at 37 °C, 150 μL of culture medium were transferred to a 96-well plate. The absorbance at 450 nm was measured by using a microplate reader (Multiskan GO, Thermo Fisher Scientific, Waltham, MA, USA). Relative cell proliferation was calculated by normalizing the absorbance value of the hypergravity exposed group to that of the static control group cultured without hypergravity exposure.

### TEER measurement

A voltohmmeter (Millicell-ERS, EMD Millipore, Billerica, MA, USA) with an Endohm-24 chamber (WPI, Sarasota, FL, USA) was used to measure TEER across RPMI 2650 cell layers. The TEER values were measured every day for 7 days to confirm the presence of a cell barrier function. In addition, TEER was measured before and after exposure to hypergravity to confirm the effect of the hypergravitational force on the perturbation of a cell barrier function. To measure the TEER at the A-L interface, 1.5 mL of culture medium were added to the apical side and then removed immediately after the TEER measurement. The measured TEER values of the RPMI 2650 cell layers were subtracted by that of a blank cup to obtain corrected TEER values. The TEER value of control group, cultured in another set of transwell inserts without hypergravity exposure, was also measured.

### Permeability assays

NaFI (MW 376 Da; Sigma), FD-4 (average MW 4000 Da; Sigma), and fluorescein-labeled jacalin (MW 66 k Da; FL-1151, Vector Laboratories, Burlingame, CA, USA) were used as markers for paracellular permeation of small polar molecules, large polar molecules, and adsorptive endocytosis, respectively. To avoid an argument against the effects of hypergravity itself on the transport rate of the marker molecules, the donor solution was added to the basolateral side instead of the apical side. Before hypergravity exposure, 2.6 mL of NaFI (25 μg/mL), FD-4 (500 μg/mL), or fluorescein-labeled jacalin (20 μg/mL) were added to the basolateral side, and 1.5 mL of culture medium were added to the apical side. After completion of the 2 hour hypergravity stimulation protocol, 100 μL of samples were taken from the apical side. The amounts of NaFI, FD-4, and fluorescein-labeled jacalin that had permeated to the apical side were determined by using a fluorescence plate reader (Varioskan LUX, Thermo Fisher Scientific) with excitation/emission wavelengths of 460/515 nm for NaFI and FD-4 and 495/520 nm for fluorescein-labeled jacalin. The *P*_*app*_ was calculated according to following equation:

1 $${P}_{app}=\frac{dQ}{dt}/({C}_{0}\times A)$$

where *dQ*/*dt* is the flux (μg/s) of the respective substances across the RPMI 2650 cell layers, *c*_0_ is the initial donor concentration (μg/mL) and *A* is the surface area (cm^2^). The *P*_*app*_ of cell-free transwell insert for each marker was also measured to determine free diffusion of each marker molecule through the porous membrane without cells.

### Immunofluorescence

Fluorescence microscopy was used to observe structural cytoskeleton changes in hNE cells following exposure to hypergravity. After completion of the 2 hour hypergravity stimulation protocol, culture media were removed and the hNE cells rinsed twice with Dulbecco’s phosphate-buffered saline (DPBS; Gibco). Samples were then fixed with 4% paraformaldehyde (Sigma) and permeabilized with 0.1% Triton X-100 in DPBS. The hNE cells were then incubated with AlexaFluor 488 conjugated phalloidin (ab176753, Abcam, Cambridge, UK) for one hour. The stained samples were then rinsed twice with DPBS and mounted with mounting solution (Vectashield H-1200, Vector Laboratories, Burlingame, CA, USA) containing DAPI for counterstaining. Fluorescence images were obtained by using an inverted microscope (CKX 53, Olympus, Tokyo, Japan) with a charge-coupled device camera (DP80, Olympus) and were processed by using cellSens software (Olympus).

### Gene expression analysis

RT-qPCR was undertaken to analyze the gravitational effects on gene expressions of junctional proteins and β-actin. After completion of the 2 hour hypergravity stimulation protocol, total RNA was extracted by using TRIzol reagent (Life Technologies, Carlsbad, CA, USA) according to the manufacturer’s protocols. The concentration of extracted RNA was measured with a microspectrophotometer (DS-11, DeNovix, Wilmington, DE, USA). Each RNA sample (1 μg) was reversely transcribed to cDNA by using a PrimeScript RT reagent kit (Takara, Shiga, Japan). The RT-qPCR was performed by using a CFX96 detection system (Bio-Rad, Hercules, CA, USA) with PerfeCTa SYBR Green SuperMix (Quanta Biosciences, Beverly, MA, USA). Target genes were CLDN-1, OCLN, ZO-1, CDH-1, and β-actin. Glyceraldehyde-3-phosphate dehydrogenase (GAPDH) was used as a housekeeping gene. Primers for the target genes are listed in Table 2. Relative mRNA expression levels were calculated with respect to GAPDH and static control group, which was cultured in another set of transwell inserts without hypergravity exposure, by using the 2^(−ΔΔCT)^ method^47^. Melting curve analysis was carried out to check for the formation of undesired PCR products such as primer dimers.

**Table 2:** Primer sequences of target genes undergoing RT-qPCR assessment.

Target gene	Size (bp^a^)	Sequences	Tm^b^
GAPDH	120	F: GAAATCCCATCACCATCTTCCAGG	61.23
		R: GAGCCCCAGCCTTCTCCATG	62.62
CLDN-1	92	F: TGGTCAGGCTCTCTTCACTGG	61.44
		R: AGGTTGTTTTTCGGGGACAGG	60.75
OCLN	115	F: CTTCCATTAACTTCGCCTGTGG	59.58
		R: TCTTTGACCTTCCTGCTCTTCC	59.96
ZO-1	76	F: ACAGCCAGCCGTTAGTCACC	62.45
		R: GTATGGGAGTTGGGGTTCATAGG	60.18
CDH-1	78	F: GCTGGACCGAGAGAGTTTCC	60.11
		R: GTTGTGCTTAACCCCTCACC	58.76
β-actin	122	F: GTCATTCCAAATATGAGATGCGTTG	59.09
		R: TGCTATCACCTCCCCTGTGT	60.25

^a^Base pair.

^b^Melting temperature (°C).

### Statistical analysis

All presented measurements were independently repeated at least three times, and the results are displayed as mean ± standard error of the mean values. A Student’s *t*-test (two-tailed, equal variance) were used to determine the statistical significance of differences between the hypergravity exposed group and the static control group.

### Data availability

All data generated or analyzed during this study are included in this published article (and its Supplementary Information files).
